# Supplementary material for: Major Adverse Cardiovascular Events in Coronary Type 2 Diabetic Patients: Identification of Associated Factors Using Electronic Health Records and Natural Language Processing
Source: J Clin Med. 2022 Oct 11;11(20):6004. doi: 10.3390/jcm11206004 (PMC9605132; doi:10.3390/jcm11206004)

Major Adverse Cardiovascular Events in Coronary Type 2 Diabetic Patients: Identification of Associated Factors Using Electronic Health Records and Natural Language Processing

SUPPLEMENTAL MATERIAL

STROBE Statement—checklist of items that should be included in reports of observational studies

	Item No.	Recommendation	Page No.	Relevant text from manuscript
**Title and abstract**	1	(*a*) Indicate the study's design with a commonly used term in the title or the abstract	1	
		(*b*) Provide in the abstract an informative and balanced summary of what was done and what was found	1	
**Introduction**				
Background/rationale	2	Explain the scientific background and rationale for the investigation being reported	2	
Objectives	3	State specific objectives, including any prespecified hypotheses	2	
**Methods**				
Study design	4	Present key elements of study design early in the paper	2	
Setting	5	Describe the setting, locations, and relevant dates, including periods of recruitment, exposure, follow-up, and data collection	3	
Participants	6	(*a*) *Cohort study*—Give the eligibility criteria, and the sources and methods of selection of participants. Describe methods of follow-up<br>*Case-control study*—Give the eligibility criteria, and the sources and methods of case ascertainment and control selection. Give the rationale for the choice of cases and controls<br>*Cross-sectional study*—Give the eligibility criteria, and the sources and methods of selection of participants	3	
		(*b*) *Cohort study*—For matched studies, give matching criteria and number of exposed and unexposed<br>*Case-control study*—For matched studies, give matching criteria and the number of controls per case	NA	
Variables	7	Clearly define all outcomes, exposures, predictors, potential confounders, and effect modifiers. Give diagnostic criteria, if applicable	3-4	
Data sources/ measurement	8*	For each variable of interest, give sources of data and details of methods of assessment (measurement). Describe comparability of assessment methods if there is more than one group	3	
Bias	9	Describe any efforts to address potential sources of bias	2-3	
Study size	10	Explain how the study size was arrived at	4	

Quantitative variables	11	Explain how quantitative variables were handled in the analyses. If applicable, describe which groupings were chosen and why	4
Statistical methods	12	(*a*) Describe all statistical methods, including those used to control for confounding	4
		(*b*) Describe any methods used to examine subgroups and interactions	NA
		(*c*) Explain how missing data were addressed	4
		(*d*) *Cohort study*—If applicable, explain how loss to follow-up was addressed<br>*Case-control study*—If applicable, explain how matching of cases and controls was addressed<br>*Cross-sectional study*—If applicable, describe analytical methods taking account of sampling strategy	NA
		(*e*) Describe any sensitivity analyses	NA
**Results**			
Participants	13*	(a) Report numbers of individuals at each stage of study—eg numbers potentially eligible, examined for eligibility, confirmed eligible, included in the study, completing follow-up, and analysed	4
		(b) Give reasons for non-participation at each stage	4
		(c) Consider use of a flow diagram	NA
Descriptive data	14*	(a) Give characteristics of study participants (eg demographic, clinical, social) and information on exposures and potential confounders	5
		(b) Indicate number of participants with missing data for each variable of interest	Suppl.
		(c) *Cohort study*—Summarise follow-up time (eg, average and total amount)	4
Outcome data	15*	*Cohort study*—Report numbers of outcome events or summary measures over time	*7*
		Case-control study—Report numbers in each exposure category, or summary measures of exposure	*NA*
		Cross-sectional study—Report numbers of outcome events or summary measures	*NA*
Main results	16	(*a*) Give unadjusted estimates and, if applicable, confounder-adjusted estimates and their precision (eg, 95% confidence interval). Make clear which confounders were adjusted for and why they were included	7
		(*b*) Report category boundaries when continuous variables were categorized	NA
		(*c*) If relevant, consider translating estimates of relative risk into absolute risk for a meaningful time period	NA

Other analyses	17	Report other analyses done—eg analyses of subgroups and interactions, and sensitivity analyses	NA
**Discussion**			
Key results	18	Summarise key results with reference to study objectives	8-10
Limitations	19	Discuss limitations of the study, taking into account sources of potential bias or imprecision. Discuss both direction and magnitude of any potential bias	10
Interpretation	20	Give a cautious overall interpretation of results considering objectives, limitations, multiplicity of analyses, results from similar studies, and other relevant evidence	10
Generalisability	21	Discuss the generalisability (external validity) of the study results	9-10
**Other information**			
Funding	22	Give the source of funding and the role of the funders for the present study and, if applicable, for the original study on which the present article is based	10

*Give information separately for cases and controls in case-control studies and, if applicable, for exposed and unexposed groups in cohort and cross-sectional studies.

Note: An Explanation and Elaboration article discusses each checklist item and gives methodological background and published examples of transparent reporting. The STROBE checklist is best used in conjunction with this article (freely available on the Web sites of PLoS Medicine at http://www.plosmedicine.org/, Annals of Internal Medicine at http://www.annals.org/, and Epidemiology at http://www.epidem.com/). Information on the STROBE Initiative is available at www.strobe-statement.org.

Table S1. Vital signs and clinical parameters of the study population at index.

	N (%)	Mean (SD)	Median (Q1, Q3)
Vital signs			
Heart rate, bpm	2415 (59.5)	66.7(24.9)	70 (59, 81)
SBP, mmHg	2423 (59.5)	140.2(22.4)	140 (125, 153)
DBP, mmHg	2424 (59.5)	74.8(13.2)	75 (67, 83)
Clinical parameters related to T2DM and CAD			
LVEF, %	590 (14.1)	50.3 (15.4)	51 (40,62)
Glucose, mg/dL	2749 (67.6)	149.5 (61.2)	135 (113,168)
HbA1c, %	1987 (48.4)	7.2 (1.5)	6.9 (6.3,7.9)
Total cholesterol, mg/dL	1943 (47.4)	165.5 (47.2)	160 (133,193)
HDL	1958 (48.4)	44.2 (15.8)	42 (35,51)
LDL	1999 (49.4)	91,4 (37.6)	85 (68, 110)
Triglycerides, mg/dL	2066 (50.5)	173.4 (253,1)	142 (99,197)
BMI, kg/m2	695 (17.1)	31.8 (7.7)	30.5 (26.9, 35.4)

DBP: Diastolic blood pressure; HbA1c: Hemoglobin A1c; HDL: High density lipoprotein; LDL: Low density lipoprotein; LVEF: Left ventricular ejection fraction; SBP: Systolic blood pressure; SD: Standard deviation. N: frequency of available data.

Table S2. Factors associated with the occurrence of MACE during the follow-up.

	**HR (CI 95%)**	**p value**
Socio-demographic characteristics		
Age, years	1.01 (<1.01, 1.02)	0.001*
Female	0.67 (0.58, 0.78)	<0.001*
Toxic habits		
History of smoking	1.32 (1.15, 1.51)	<0.001*
Current smoker	0.98 (0.83, 1.17)	0.849
Former smoker	1.34 (1.17, 1.53)	<0.001*
Clinical characteristics		
**T2DM**		
Non-insulin-dependent	0.76 (0.66, 0.86)	<0.001*
Insulin-dependent	1.32 (1.16, 1.51)	<0.001*
Time since first mention of T2DM in EHRs, years	1.01 (<1.01, 1.02)	0.041*+
Age at first mention of T2DM in EHRs, years	<1.01 (>0.99, 1.01)	0.063
**CAD**		
Single coronary vessel disease	1.09 (0.93, 1.29)	0.277
Multivessel coronary disease	2.50 (2.18, 2.87)	<0.001*
Left main coronary artery disease	2.10 (1.16, 3.81)	0.014*
Time since first mention of CAD in EHRs, years	1.02 (1.01, 1.04)	0.002*
Age at first mention of CAD in EHRs, years	1.01 (<1.01, 1.01)	0.038*+
Comorbidities		
**Blood and lymphatic system disorders**		
Anemia	0.99 (0.83, 1.16)	0.861
**Cardiovascular disorders**		
Transient Ischemic Attack	2.35 (1.70, 3.24)	<0.001*
Arterial hypertension	1.29 (1.05, 1.58)	0.015*
Heart Failure	1.43 (1.23, 1.65)	<0.001*
Atrial Flutter	1.21 (1.03, 1.43)	0.024*
Atrial fibrillation	1.21 (1.01, 1.45)	0.034*+
Heart Valve Disease	1.33 (1.16, 1.52)	<0.001*
Peripheral Vascular Disease (PVD)	1.35 (1.18, 1.55)	<0.001*
Peripheral Artery Disease (PAD)	1.66 (1.40, 1.98)	<0.001*
Angina	1.85 (1.61, 2.11)	<0.001*

Unstable angina	1.72 (1.49, 1.99)	<0.001*
Stable angina	1.53 (1.31, 1.78)	<0.001*
**Endocrine, metabolism, and nutrition disorders**		
Hyperlipidemia	0.98 (0.86, 1.12)	0.778
Hypoglycemia	1.12 (0.83, 1.51)	0.463
Gout	1.18 (0.86, 1.62)	0.298
Hyperthyroidism	1.14 (0.72, 1.82)	0.574
Hypothyroidism	0.76 (0.59, 0.98)	0.037*+
Obesity	0.96 (0.83, 1.11)	0.554
**DM-related disorders**		
Diabetic retinopathy	1.43 (1.13, 1.81)	0.003*
Diabetic foot	1.47 (0.76, 2.84)	0.250
Diabetic neuropathy	1.17 (0.81, 1.69)	0.410
**Gastrointestinal and hepatobiliary disorders**		
Chronic liver disease	0.87 (0.54, 1.41)	0.580
**Psychiatric disorders**		
Depression/Anxiety	0.95 (0.80, 1.13)	0.579
**Renal and urinary disorders**		
CKD	0.93 (0.78, 1.11)	0.444
**Respiratory/mediastinal disorders**		
COPD/Asthma	1.34 (1.14, 1.59)	<0.001*
Sleep apnea	0.98 (0.80, 1.20)	0.836
**Treatments**		
**Insulin treatment**	1.51 (1.31, 1.74)	<0.001*
Fast-acting	1.20 (0.96, 1.50)	0.117
Intermediate-acting	1.42 (0.99, 2.03)	0.057
Intermediate- or long-acting combined with fast-acting	1.55 (1.21, 1.99)	<0.001*
Long-acting	1.38 (1.18, 1.62)	<0.001*
**Oral hypoglycemic agents**		
Metformin	1.08 (0.92, 1.28)	0.339
iDPP4	1.07 (0.91, 1.26)	0.403
iSGLT2	1.18 (0.75, 1.83)	0.473
GLP1-RA	0.90 (0.65, 1.24)	0.517
Sulfonylureas	1.23 (1.05, 1.43)	0.008*
Thiazolidinediones	1.21 (0.81, 1.82)	0.351
Alpha-glucosidase	0.45 (0.20, >0.99)	0.049*+
Glinides	0.94 (0.77, 1.16)	0.584
Combination therapies	1.10 (0.94, 1.28)	0.227
**Anticoagulant therapy**	1.21 (1.03, 1.42)	0.021*
Vitamin k antagonist oral anticoagulant	1.14 (0.95, 1.37)	0.167
Warfarina	2.20 (0.82, 5.87)	0.117
Acenocumarol	1.14 (0.95, 1.38)	0.160
Nonvitamin k antagonist oral anticoagulant	1.28 (1.04, 1.56)	0.017*
Heparin group	1.37 (1.09, 1.71)	0.006*
Direct thrombin inhibitors	0.81 (0.45, 1.47)	0.490
Direct factor Xa inhibitors	1.66 (1.09, 2.54)	0.019*
Fondaparinux	2.21 (0.99, 4.94)	0.053
**Antiplatelet agents**	2.16 (1.80, 2.59)	<0.001*
ASA	1.83 (1.56, 2.15)	<0.001*
Clopidogrel	1.75 (1.53, 2.00)	<0.001*
Prasugrel	1.89 (1.26, 2.83)	0.002*
Ticagrelor	1.44 (0.95, 2.18)	0.085
Other antiplatelet agents	1.41 (1.14, 1.76)	0.002*
DAPT	1.70 (1.48, 1.95)	<0.001*
Clopidogrel + ASA	1.67 (1.44, 1.93)	<0.001*
Prasugrel + ASA	1.78 (1.12, 2.84)	0.015*
Ticagrelor + ASA	1.62 (1.02, 2.59)	0.043*+
**Other pharmacological treatments**	1.43 (0.93, 2.21)	0.103
Beta Blockers	1.53 (1.32, 1.78)	<0.001*

ACE Inhibitors or ARB	1.17 (0.98, 1.40)	0.091
ACE Inhibitors	1.09 (0.95, 1.25)	0.197
ARB	1.08 (0.95, 1.24)	0.249
Calcium channel Blockers	1.31 (1.15, 1.50)	<0.001*
Nitrates	1.70 (1.48, 1.94)	<0.001*
Ivabradine	1.39 (1.08, 1.78)	0.009*
Ranolazines	1.84 (1.46, 2.30)	<0.001
Diuretics	1.19 (1.04, 1.36)	0.012*
Lipid lowering drugs	1.41 (1.15, 1.73)	0.001*
Statins	1.32 (1.10, 1.58)	0.003*
Other lipid lowering drugs	1.10 (0.94, 1.29)	0.215
**Revascularization treatments**	3.23 (2.76, 3.78)	<0.001*
PCI	3.09 (2.69, 3.56)	<0.001*
CABG	1.56 (1.33, 1.84)	<0.001*
Time since last-mentioned PCI in EHRs (years)	1.01 (0.99, 1.04)	0.417
Time since last-mentioned CABG in EHRs (years)	0.97 (0.95, >0.99)	0.035*+

ACE: Angiotensin-converting enzyme; ARB: Angiotensin receptor blockers; ASA: Acetyl salicylic acid; CABG: Coronary artery bypass graft; CAD: Coronary artery disease; CHD; Coronary heart disease; CI: Confidence interval; CKD: Chronic kidney disease; COPD: Chronic obstructive pulmonary disease; DAPT: Dual antiplatelet therapy<, EHR: Electronic health records; PAD: Peripheral artery disease; GLP1-RA: Glucagon-like peptide 1 receptor agonist; iDPP4: Dipeptidyl peptidase-4 inhibitor; iSGLT2: Sodium-glucose co-transporter 2 inhibitor; PCI: Percutaneous coronary intervention; PVD: Peripheral vascular disease; T2DM: Type 2 diabetes mellitus.

*Statistical differences were considered significant when p < 0.05 in two-tailed test. +When correcting for multiple testing (adjusted p values), p > 0.05.

Figure S1. Probability of any new MACE (myocardial infarction, stroke, unstable angina, and urgent revascularization) is shown. The number of patients at risk across the follow-up period is indicated below.

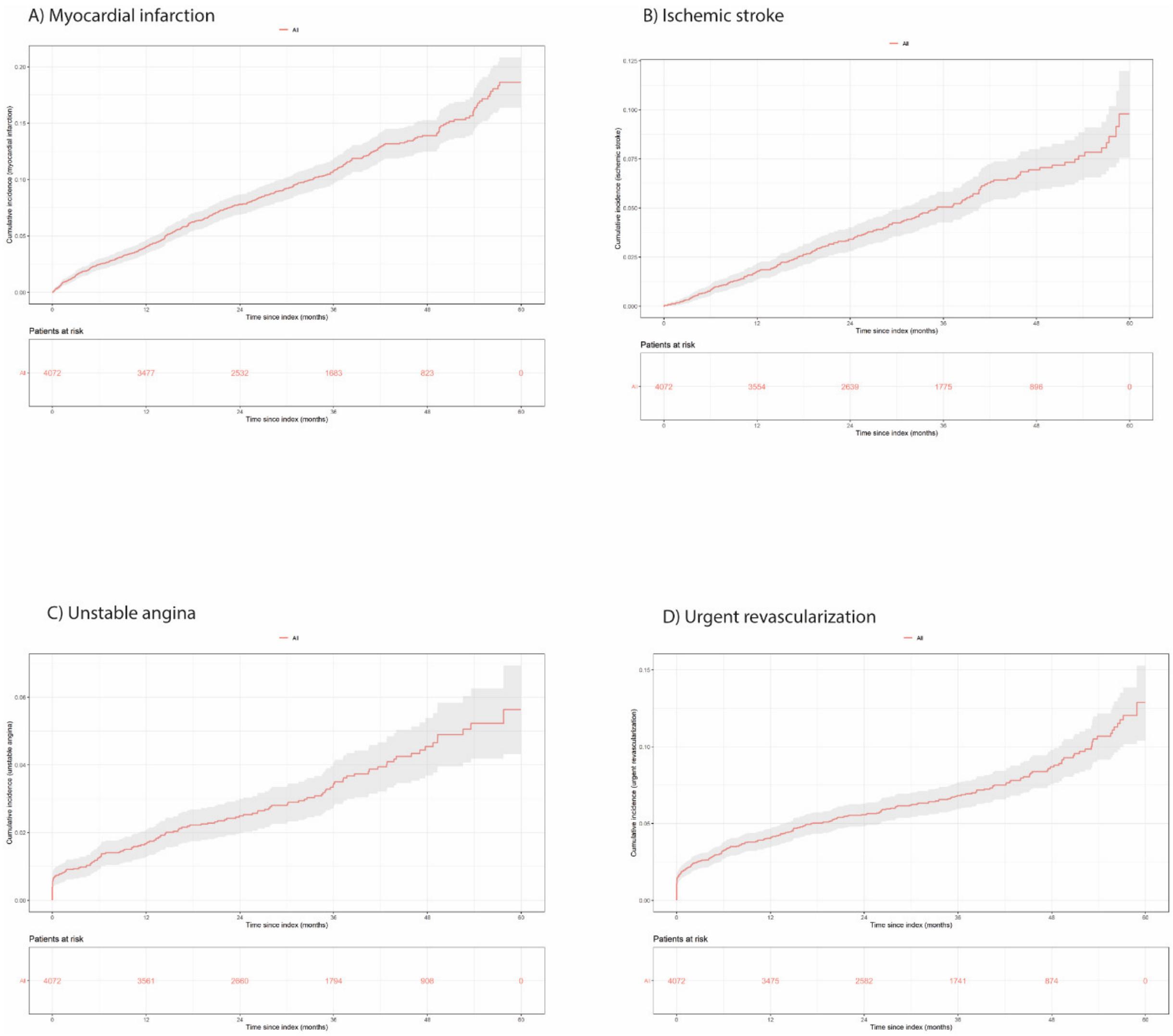

Supplement: Supplementary file 1 [file jcm-11-06004-s001.zip › jcm-1932868-supplementary.pdf]
